# Vibrational Spectroscopy Saliva Profiling as Biometric Tool for Disease Diagnostics: A Systematic Literature Review

**DOI:** 10.3390/molecules25184142

**Published:** 2020-09-10

**Authors:** Stéphane Derruau, Julien Robinet, Valérie Untereiner, Olivier Piot, Ganesh D. Sockalingum, Sandrine Lorimier

**Affiliations:** 1 Université de Reims Champagne-Ardenne, Département de Biologie Orale, UFR Odontologie, 2 rue du Général Koenig, 51100 Reims, France; stephane.derruau@univ-reims.fr (S.D.); julien.robinet@etudiant.univ-reims.fr (J.R.); 2 Pôle de Médecine Bucco-dentaire, Centre Hospitalier Universitaire de Reims, 45 rue Cognacq-Jay, 51092 Reims, France; 3 Université de Reims Champagne-Ardenne, BioSpecT-EA7506, UFR de Pharmacie, 51 rue Cognacq-Jay, 51097 Reims, France; olivier.piot@univ-reims.fr (O.P.); ganesh.sockalingum@univ-reims.fr (G.D.S.); 4 Université de Reims Champagne-Ardenne, PICT, 51 rue Cognacq-Jay, 51097 Reims, France; valerie.untereiner@univ-reims.fr; 5 Université de Reims Champagne-Ardenne, GRESPI-EA4694, UFR Sciences Exactes et Naturelles, 51687 Reims, France

**Keywords:** saliva, vibrational spectroscopy, Raman, infrared, diagnosis, systematic review

## Abstract

Saliva is a biofluid that can be considered as a “mirror” reflecting our body’s health status. Vibrational spectroscopy, Raman and infrared, can provide a detailed salivary fingerprint that can be used for disease biomarker discovery. We propose a systematic literature review based on the PRISMA (Preferred Reporting Items for Systematic Reviews and Meta-Analyses) guidelines to evaluate the potential of vibrational spectroscopy to diagnose oral and general diseases using saliva as a biological specimen. Literature searches were recently conducted in May 2020 through MEDLINE-PubMed and Scopus databases, without date limitation. Finally, over a period of 10 years, 18 publications were included reporting on 10 diseases (three oral and seven general diseases), with very high diagnostic performance rates in terms of sensitivity, specificity, and accuracy. Thirteen articles were related to six different cancers of the following anatomical sites: mouth, nasopharynx, lung, esophagus, stomach, and breast. The other diseases investigated and included in this review were periodontitis, Sjögren’s syndrome, diabetes, and myocardial infarction. Moreover, most articles focused on Raman spectroscopy (*n* = 16/18) and more specifically surface-enhanced Raman spectroscopy (*n* = 12/18). Interestingly, vibrational spectroscopy appears promising as a rapid, label-free, and non-invasive diagnostic salivary biometric tool. Furthermore, it could be adapted to investigate subclinical diseases—even if developmental studies are required.

## 1. Introduction

Translation of precision medicine into mainstream clinical care is being prioritized worldwide and is increasingly being advanced as the future paradigm for more effective medical management. Precision medicine, also coined as P4 medicine by Hood and Friend [1], who characterized it as being “predictive”, “preventive”, “personalized”, and “participatory”, embraces a system approach to understanding underlying disease pathophysiology coupled with individually tailored healthcare informed by an individual’s genes, lifestyle, and environment [2,3]. The search for biomarkers can then be beneficial in various clinical situations for patient management: Screening of patients at risk of the disease or with the disease at an early stage, differential diagnosis of the disease with other conditions, the prognosis of the disease independently of the treatment, prediction of the response to treatment, and monitoring of disease evolution [4].

In this context of the search for diagnostic markers, vibrational spectroscopy (VS), infrared absorption (IR), and Raman scattering spectroscopies, appears to be a promising alternative approach in research for developing new modalities with the aim to improve patient healthcare via the better diagnosis, prognosis, and surveillance. vs. modalities hold such promises because the ‘‘molecular fingerprint’’ that it provides a snapshot of the sample biomolecular composition, and variations therein can be exploited to identify disease status [4]. The diagnostic potential of vs. approach has been published. However, in the vast majority of cases as proof-of-concept studies, mainly in malignant tumors on various types of biosamples, such as biofluids [5], cells [6], or tissues [7]. Biofluids seem particularly suitable for the detection of many types of diseases, because they are in direct connection with organs of the human body and are generally easily collected [4,8].

Saliva (or whole saliva or oral fluid) is an interesting biofluid. It is a biological fluid composed of secretions from the three major salivary glands (parotid, submandibular, sublingual) and from minor glands (e.g., labial, buccal, lingual, and palatal tissues), gingival crevicular fluid, cell debris, dental plaque, bacteria, nasal and bronchial secretions, flaking cells, blood and exogenous substances [9]. Total human saliva is a biofluid, constituted by water (99.5%), proteins (0.3%, e.g., proline-rich, tyrosine-rich, mucins, sialic acid, lactic acid), and inorganic substances (0.2%, e.g., thiocyanate) reflecting the physiological and pathological state of the body [9,10,11]. Saliva includes over 800 identified metabolites (amino acids, carboxylic acids, steroid derivatives, glucose, etc.) and is comparable to human serum metabolomes in terms of chemical complexity and abundance of metabolites [9,12].

In a healthy individual, the daily salivary secretion is estimated to be between 0.5 and 1.5 L. Its collection is easy, non-invasive, painless, and low-cost with minimal risks of exposure to infectious agents [13]. Studies with different techniques of proteomics, metabolomics, transcriptomics, or microbiomics have shown the potential interest of using saliva in the diagnosis of oral diseases (such as periodontitis or oral cancer), but also of systemic diseases (such as breast cancer, diabetes, and Sjögren’s syndrome) [9,13].

Thus, the aim of this systematic literature review was to demonstrate the real potential of vs. to diagnose oral and general diseases using saliva. Literature searches were recently conducted without date limitation, in May 2020, through MEDLINE-PubMed and Scopus databases, according to PRISMA (Preferred Reporting Items for Systematic Reviews and Meta-Analyses) guidelines.

## 2. Results

The initial search using keywords combinations returned 172 articles on PubMed and 164 articles on Scopus. In the first phase, duplicates articles were removed. Titles and abstracts of the remaining 267 papers were reviewed, and 227 of them were excluded as they were not relevant to the inclusion criteria. The remaining full-text articles have been thoroughly scrutinized, and one additional article was found after scrutinizing the references of these 40 retained papers.

**Figure 1:**
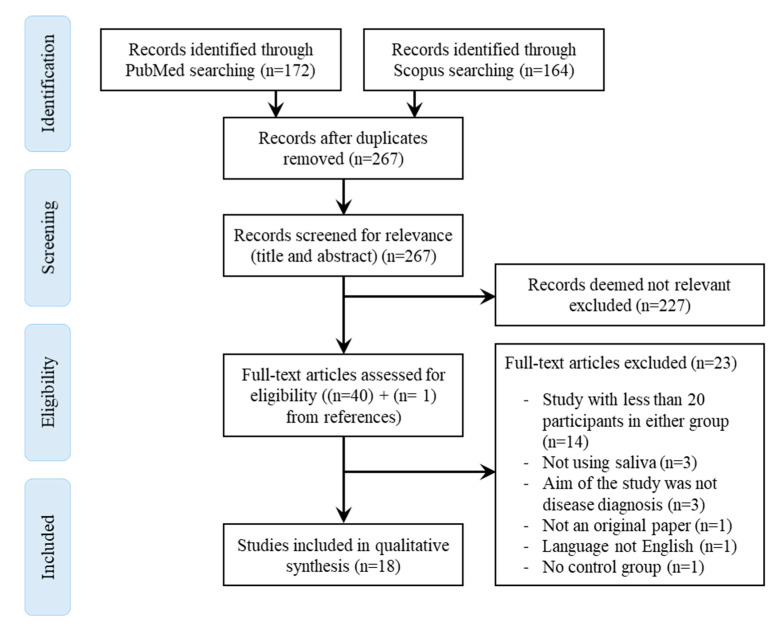
PRISMA (Preferred Reporting Items for Systematic Reviews and Meta-Analyses) diagram for the selection of relevant studies.

All 41 articles were assessed for eligibility, and 23 were finally excluded. The reasons for excluding these full-text articles were because they had less than 20 patients in either group (*n* = 14), they did not use saliva (*n* = 3), the aim of the study was not the diagnosis of a disease (*n* = 3), it was not an original document (*n* = 1), not written in English (*n* = 1) or because there was no control group (*n* = 1). Finally, 18 key papers have been included in this systematic literature review (Figure 1).

**Figure 2:**
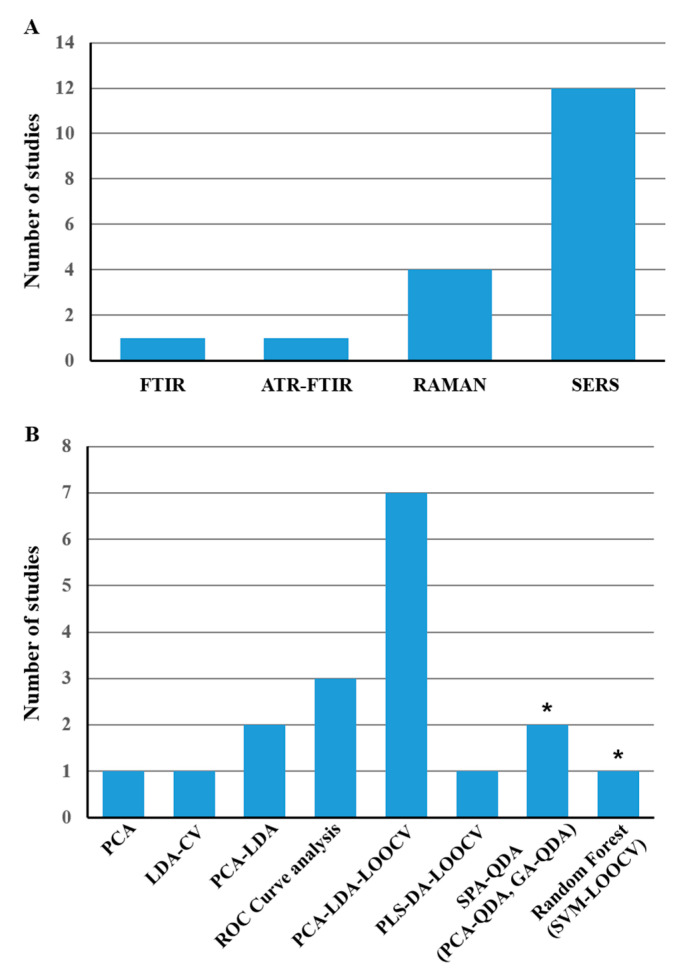
The number of studies, according to vibrational spectroscopy (VS) and algorithms techniques. (**A**) Vibrational spectroscopic techniques; (**B**) Algorithms used for data processing; * indicates results from the most relevant algorithm according to the authors; PCA, principal component analysis; LDA, linear discriminant analysis; LOOCV, leave-one-out cross-validation; SPA-QDA, successive projections algorithm quadratic discriminant analysis; GA-QDA, genetic algorithm quadratic discriminant analysis; PCA-QDA, principal component analysis quadratic discriminant analysis; SVM, support vector machine; PLS-DA, partial least squares discriminant analysis; ROC, receiver operating characteristics.

Although this review aimed to investigate the use of vibrational spectroscopies (Infrared and Raman) as a tool for disease diagnostics, most articles were interested in using Raman spectroscopy (*n* = 16/18), and more, specifically surface-enhanced Raman spectroscopy (SERS) (*n* = 12/18). Only two articles focused on Fourier Transform Infrared Spectroscopy (FTIR). This is summarized in Figure 2A. The diagnostic performances in terms of sensitivity, specificity, and accuracy were evaluated by different algorithms. From Figure 2B, it can be noticed that the model based on PCA-LDA followed by LOOCV was most widely used.

Among those 18 articles, 10 different diseases were studied: Three oral and seven general diseases. Moreover, cancers remain the most studied pathology with 13 articles out of 18. The total number of patients included in studies of this review was 2082 with 1226 patients with diagnosed diseases (*n* = 925) or premalignant disorders/intermediate stage (*n* = 301) and 856 healthy volunteers (Table 1).

### 2.1. Cancer

Cancer represents the main disease for which vs. has been used as saliva-based diagnostic tool. Thirteen articles included in this review focused on six different cancers: oral (*n* = 2), nasopharyngeal (*n* = 3), lung (*n* = 2), esophageal (*n* = 2), gastric (*n* = 1), and breast (*n* = 3) (Table 1).

The total number of patients, including in these cancer studies was 1736 out of which 747 were with cancer (82 squamous cell carcinoma, 264 nasopharyngeal cancer, 82 lung cancer, 49 esophageal adenocarcinoma, 104 gastric cancer, and 166 breast cancer), 271 patients were with premalignant disorders (115 oral, 123 esophageal, and 33 breast lesions) and 718 were healthy patients.

#### 2.1.1. Oral Cancer

Saliva is directly in “contact” with oral squamous cell carcinoma, the most common oral malignancy. In 2016, Jaychandran et al. presented a study based on conventional Raman spectroscopy, evaluating saliva for discrimination of oral squamous cell carcinoma (50 patients), compared to oral premalignant disorders (87 patients) and healthy controls (21 patients) [14]. They also compared their results on saliva with other ‘liquid biopsies’ (blood and urine) and a conventional tissue biopsy. Spectroscopic data analysis was through a principal component analysis (PCA) followed by linear discriminant analysis (LDA). Raman peaks for discrimination between malignant, premalignant, and normal groups were observed for pyrimidine, amide, mucin, hemocyanin, and carotenoids (Table 2). Results showed that PCA-LDA was able to discriminate spectra from cancer patients versus non-cancer with an accuracy of 93.1%. Moreover, accuracy was better with saliva samples than with blood or urine (78% and 90.5%, respectively), with however best results obtained with tissue samples. Mean sensitivities and specificities were not described.

Rekha et al. performed a study, also using Raman and PCA-LDA, but with a leave-one-out cross-validation (LOOCV) [15]. They were interested only in saliva sample analysis and compared samples of 23 healthy volunteers to both, 28 patients with oral submucous fibrosis (premalignant group), and 32 clinically diagnosed patients for oral squamous cell carcinoma.

Raman peaks showing differences between different patient groups corresponded to various amino acids, such as histidine, valine, and proline, as well as amide I, nucleic acid, lactic acid, and lipids (Table 2).

The predictive model based on PCA-LDA showed that it could correctly classify spectra from cancer (malignant) against non-cancer (normal) groups with a sensitivity, specificity, and accuracy of 93.8%, 82.6%, and 89.1%, respectively. Interesting results were also obtained upon comparing healthy and precancerous lesion samples (premalignant): 96.4% of sensitivity, 70.2% of specificity, and 84.3% of accuracy. However, the comparison of the three groups (normal, premalignant, and malignant) simultaneously resulted in a classification accuracy of only 60.2%.

#### 2.1.2. Nasopharyngeal Cancer

Other cancers can contribute endogenously to the composition of saliva via the nasal and bronchial secretions, such as cancers of the nasopharynx or lung. Nasopharyngeal carcinoma (NPC) is by far the most common cancer in the nasopharynx cancers [32].

Feng’s group published three articles between 2014 and 2017 on this subject. In the first, Feng et al. carried out a SERS analysis on purified proteins from saliva samples from 62 patients with diagnosed nasopharyngeal cancer and from 30 healthy donors [16]. They used the PCA-LDA model with LOOCV. The authors attribute the SERS peaks of discrimination to phenylalanine, tyrosine, tryptophan, proline, certain proteins, collagen, phospholipids, and Amide I (Table 2). The use of a ROC (receiver operating characteristic) curve allowed them to obtain with this model an AUC (area under curve), providing some measure of aggregate classification performance. AUC was of 92.4%, as well as sensitivity, specificity, and accuracy values of 98.4%, 73.3%, and 90.2%, respectively, indicating the approach to be a promising one.

In 2016, Qiu et al. presented a complementary study with an identical protocol (SERS analysis, spectral processing with PCA-LDA-LOOCV, and calculation of a ROC curve) and a similar population (32 patients with nasopharyngeal carcinoma *versus* 30 non-cancer volunteers) [17]. The only difference is that the analysis was done on the whole saliva of the patients without prior purification of the proteins. Major differences in peak intensities between the cancer group and the control group were highlighted. These peaks were attributed, among others, to adenine, nucleic acids, collagen, phenylalanine, glycogen, and fatty acids (Table 2). The results obtained here were very slightly lower than the previous ones: The AUC of the ROC curve was 91.8, and the classification accuracy was 83.9% for a sensitivity of 86.7%, and a specificity of 81.3%.

In 2017, the same group, Lin X. et al., published another report, with a bigger cohort (170 patients with nasopharyngeal carcinoma and 71 controls) [18]. Moreover, the spectral analysis was performed on the purified saliva proteins, and the rest of the protocol remained strictly identical. Specific SERS peaks were also identified between cancer and control groups, particularly corresponding to phenylalanine, proline, valine, proteins, and collagens (see Table 2). The performance of the prediction model was, however, less than with the two previous studies, with an AUC of the ROC curve of 0.795, as well as sensitivity, specificity, and classification accuracy of 70.7%, 70.3%, and 70.5%, respectively.

#### 2.1.3. Lung Cancer

In 2012, Li et al. used SERS on saliva samples taken from 21 clinically diagnosed lung cancer patients and from 20 healthy [19]. Major changes regarding peaks between these two groups were assigned to amino acids and nucleic acid bases (Table 2). After multivariate analysis with PCA combined with LDA, the study resulted in sensitivity, specificity, and accuracy of 78%, 83%, and 80%, respectively.

Qian et al. carried out in 2018, a study using SERS to discriminate 61 lung cancer saliva samples from 66 non-cancer controls [20]. Twelve peaks that varied significantly from one group to another were identified and attributed mainly to change in protein residues and the content of nucleic acid molecules (Table 2). Chemometrics analysis was performed using two algorithms: Support vector machine (SVM) and random forest (RF). Differences in SVM results between lung cancer patients and healthy participants’ saliva were highlighted after a LOOCV. Slightly better results were achieved with the RF method, reporting an optimal sensitivity and specificity of 96.7% and 100%, respectively, although the SVM method was not that outdone, with a sensitivity of 95.1% and a specificity of 100%.

#### 2.1.4. Esophageal Cancer

Furthermore, other cancers, such as esophageal and gastric, can contribute endogenously to the composition of saliva by the gastroesophageal reflux. Maitra et al. published two articles in 2019 and 2020 about esophageal adenocarcinoma, which is the most common esophageal cancer in the developed world [21,22]. In these studies, they collected samples of four different biofluids (plasma, serum, urine, and saliva) from six categories of patients: Patients with a diagnosed esophageal adenocarcinoma (OAC), with high grade dysplasia (HGD), with low grade dysplasia (LGD), with Barrett’s esophagus (a premalignant lesion of esophageal adenocarcinoma), with esophageal inflammation, and healthy volunteers. The two studies differed by the techniques used, attenuated total reflectance-Fourier transform infrared spectroscopy (ATR-FTIR) for the first, and conventional Raman spectroscopies for the second. Several predictive models were built using different supervised classification algorithms (principal component analysis quadratic discriminant analysis, PCA-QDA; successive projections algorithm quadratic discriminant analysis, SPA-QDA; genetic algorithm quadratic discriminant analysis, GA-QDA).

With ATR-FTIR, the best results were achieved with SPA-QDA [21]. Category-distinguishing wavenumbers obtained for SPA-QDA and GA-QDA models corresponded to regions of phosphodiester, polysaccharides, pectin, phosphate II, phenyl vibrations, amide I, guanine, and lipids (Table 2). SPA-QDA allowed to correctly classify the different categories of patients, and in particular those greater than 20 patients: Normal (*n* = 38), Barrett’s esophagus (*n* = 27) and OAC (*n* = 25) with accuracy values between 88.8% and 96.3%. With SPA-QDA, the value of sensitivity was between 95.4% and 100%, and the value of specificity was between 62.5% (healthy) and 100% (Table 1).

Raman spectroscopy was not outdone, since the most efficient predictive model (SPA-QDA) had a sensitivity of 100% (for all groups greater than 20 patients, i.e., normal with 35 patients, Barrett’s with 26, OAC with 24) and a specificity between 80% (Barrett’s) et 100% (OAC) [22]. Accuracy values were between 95.6% and 100%. Discriminant wavenumbers obtained for SPA-QDA or GA-QDA models can be assigned to regions of phosphodiester, ribose, phenyl vibrations, nucleic acids, amide I, guanine, thymine, and lipids (Table 2).

#### 2.1.5. Gastric Cancer

Chen et al. [23] performed a study using SERS on saliva samples of 84 late gastric cancer, 20 early gastric cancer, and 116 healthy volunteers. They relied on studies that demonstrated that certain metabolites, such as amino acids could be used as cancer biomarkers [33,34]. They first carried out a saliva assay of the 10 most concentrated amino acids in saliva (taurine, glycine, glutamine, ethanolamine, histidine, alanine, glutamic acid, hydroxylysine, proline, and tyrosine) and whose concentrations varied the most between the three categories of patients (Table 2). They found using a ROC curve that the combination of 10 amino acids allowed them to distinguish patients with gastric cancer from healthy patients. After this first step, they developed a protocol to detect these amino acids with SERS, before using it on the saliva samples. Spectroscopic data analysis was performed using PCA to discriminate the spectra of the patients according to the different groups. With this approach, they achieved a sensitivity of 87.7% and a specificity of 80% in discriminating advanced gastric cancer from non-cancer patients. The results concerning early gastric cancer were not to be outdone, with a sensitivity and a specificity of 80.0% and 88.8%, respectively. Mean accuracy was not described, but a negative predictive value of 94.5% was given for controls [23].

#### 2.1.6. Breast Cancer

Three studies have investigated breast cancer, although saliva is not in direct contact with this organ.

The first study on breast cancer screening using vs. of saliva samples was published in 2015 by Feng et al. [24]. They included 31 patients with proven cancer, 33 patients with a benign tumor, and 33 healthy volunteers. Spectral analysis was performed after saliva protein purification, and the main observed peaks were attributed to phenylalanine, tryptophan, tyrosine, hydroxyproline, proline, amide I and III, collagen, and lipids (Table 2). PLS-DA, a regression extension of PCA, was employed in combination with LOOCV to analyze and discriminate between the saliva protein SERS spectra of the three groups of participants. The authors achieved a sensitivity between 72.7% and 75.8%, a specificity between 81.3% and 93.8%, and an overall accuracy between 78.4% and 87.6% to discriminate breast cancer, benign breast lesions, and control (Table 1).

In 2017, Hernández-Arteaga et al. published a study on breast cancer diagnosis based on SERS of salivary sialic acid assay [25] by considering the previous observations by Ozturk et al., who demonstrated that sialic acid concentration was significantly higher in breast cancer patients than in control patients [35]. Using a calibration set of sialic acid (SA), the concentration of SA correlated well with three peak intensities at 1002, 1237, and 1391 cm^−1^ corresponding to pyranose, amide III, and carboxyl, respectively (Table 2). Results showed that salivary SA concentration was significantly higher in the cancer group (*n* = 100) compared to the control group (*n* = 106), 18.5 mg/dL and 3.5 mg/dL, respectively. Moreover, the authors concluded that the discriminating threshold concentration of sialic was 7 mg/dL. This method showed a sensitivity of 94%, a specificity of 98%, and an accuracy of 92%.

In 2019, the same group conducted a similar SERS study with 35 breast cancer patients and 129 healthy patients [26]. ROC curve defines a threshold value of salivary SA of 12.5 mg/dL, for which sensitivity and specificity were 80.6% and 93.1%, respectively. In addition, the Area Under Curve (AUC) calculated from the ROC curve at this sialic acid concentration reached 0.95, indicating that this diagnostic test is very promising.

### 2.2. Other Diseases

Among the 18 selected articles, five dealt with four diseases unrelated to cancer, two concerning oral diseases directly “in contact” with saliva (periodontitis and Sjögren’s syndrome) In contrast, two others were systemic diseases (diabetes and myocardial infarction).

#### 2.2.1. Periodontitis

Periodontitis are multifactorial infectious diseases affecting almost 50% of the population with an inflammatory component. They affect supporting tissues of the tooth and can lead to tooth loss in their most advanced stages [36].

In 2019, Hernandez-Cedillo et al. reported a study using SERS [27] for discriminating periodontitis from control samples. This work aimed at carrying out a dosage of sialic acid in the saliva of 93 subjects: Thirty-three with periodontitis, thirty with gingivitis (superficial periodontal inflammation), and thirty healthy volunteers. For this purpose, they used the same protocol they had developed in 2017 for breast cancer [25]. Their results showed that patients with periodontitis could be discriminated from healthy volunteers, but not from those with gingivitis. Moreover, they determined a “threshold” concentration of 12 mg/mL of sialic acid above which the diagnosis of periodontitis could be performed. For this concentration, the test had a sensitivity of 69.6%, a specificity of 100%, and the AUC (ROC curve) was 88.8%.

#### 2.2.2. Sjögren’s Syndrome

Sjögren’s syndrome (SjS) is a systemic autoimmune disease that is characterized by lymphoid infiltration of the salivary and lacrimal glands [28]. In 2019, Stefancu et al. used SERS of saliva and serum samples to discriminate SjS (*n* = 29) from control (*n* = 21) subjects [28]. For both saliva and serum, SERS spectra depicted some similar bands that were attributed mainly to purine metabolites, such as uric acid, xanthine, and hypoxanthine (Table 2). The supervised classification model based on PCA-LDA followed by LOOCV resulted in a sensitivity, specificity, and accuracy of 96.5%, 90.5%, and 94% for saliva and 96.5%, 100%, and 98% for serum, respectively. In 2020, the same group, Moisoiu et al., conducted a SERS analysis on saliva from SjS (*n* = 31) and control (*n* = 22) subjects [29]. Spectral analysis and processing remained strictly identical. Mean sensitivity and specificity were 77% and 74%, respectively, whereas the overall accuracy was 75%. A distinguishing feature of this study was the association of SERS with a two-dimensional ultrasonic elastography technique that improved sensitivity, specificity, and accuracy to 80%, 81%, and 81%, respectively.

#### 2.2.3. Diabetes

Diabetes is a multifactorial metabolic disease characterized by chronic hyperglycemia and disturbances in the metabolism of carbohydrates, lipids, and proteins. It is caused by a deficiency in insulin secretion (type I), the action of insulin (type II), or both [30,37,38].

The only publication on the saliva-based diagnosis of diabetes dates from 2010. In this work, Scott et al. performed an FTIR analysis of saliva samples from 39 diabetic patients and 22 control patients [30]. An LDA was employed to identify six discriminant spectral regions that best differentiate diabetic from control patients attributable to glycation products, proteins, and amino acids (Table 2). An accuracy of 88.2% was obtained with LDA-cross validation on the test set. Sensitivity and specificity values were not provided.

#### 2.2.4. Acute Myocardial Infarction

Acute myocardial infarction (AMI) is a myocardial necrosis caused by ischemia and persistent hypoxia related to obstruction of a coronary artery [39].

In 2015, Cao et al. studied saliva samples from 46 AMI patients and 43 healthy volunteers [31]. A conventional Raman spectroscopy analysis was performed, and data were processed by PCA-LDA, followed by a LOOCV. Prominent Raman peaks were identified and assigned at cysteine, phenylalanine, tyrosine, tryptophan, hydroxyproline, nucleic acids, proteins, amide I and II (Table 2). A ROC curve was constructed from the results obtained using the predictive model. The sensitivity and specificity were 80.4% and 81.4%, respectively, while the calculated AUC was 0.855. This study suggested Raman spectroscopy as a potential diagnostic tool.

**Figure 3:**
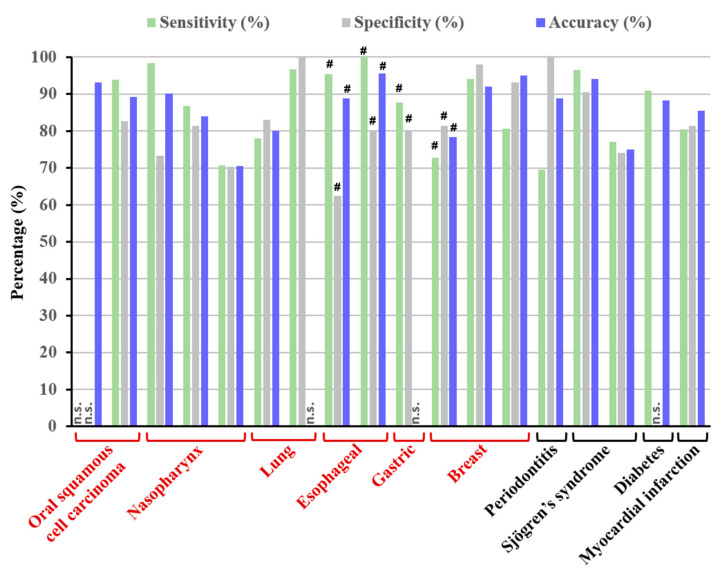
Percentage of sensitivity, specificity, and accuracy of selected studies. # indicates the minimal result according to the different categories of patients; red indicates diseases corresponding to cancers; n.s., not stated.

For a visual comparison and in order to summarize the diagnostic performances of the different prediction models used in the 18 studies concerning vs. of saliva, the values are displayed in Figure 3.

## 3. Discussion

In this work, we assessed the potential of vibrational spectroscopy as a biometric tool to diagnose oral and general diseases using saliva as a biological specimen. This systematic literature review was conducted using MEDLINE-PubMed and Scopus databases, according to the PRISMA guidelines.

The selected studies show the promising potential of saliva-based vibrational spectroscopy as a non-invasive and rapid diagnostic tool. The majority of researches in the field of biomedical vibrational spectroscopy has been based on the analysis of human tissues, healthy and cancerous tissues, such as breast, lung, colon, prostate, oral, liver, kidney, and many others. In the past decade, there has been growing interest in a multiple of blood-derived biofluids, also termed “liquid biopsies”, such as serum and plasma, but also bile, urine, and saliva because of their availability in comparison with solid biopsies. Indeed, 267 publications have been identified as using vs. of saliva for diagnostics, but a large majority of them are pilot or proof-of-concept studies, including a small number of patients.

It is important to note that specific characteristics as disease prevalence (impacting positive and negative predictive values) and sample numbers are important in order to evaluate the clinical utility of this tool. Hence, in the context of this study, we defined a minimum of 20 patients per group as one of the criteria according to a report by Bell et al. [40], who showed in 2018 that with this number, the size effect seems to have a limited impact (small standardized difference) on the results for pilot studies compared to the main trial. In addition, the size effect has more impact below *n* = 20 as the sensitivity and specificity values were very random, although the essential statistical parameters were considered in the evaluation for clinical utility. Consequently, other vs. studies using saliva, were not included in this review, due to a number of patients less than 20 per group, and concerning other diseases, such as burning mouth syndrome [41], asthma [42], Alzheimer’s disease [43], chronic renal failure [44], ovarian cancer [45], various infections, e.g., influenza [46] or pseudomonas [47], and cystic fibrosis [48]. Therefore, out of the 267 studies, only 18 satisfied the criteria according to the PRISMA guidelines.

In this review, results from all selected publications were very promising with interesting accuracy values of 70–80% for three studies, 80–90% for five studies, and >90% for five studies. Five studies presented no accuracy value. It is noteworthy that for both cancer and non-cancer pathologies, the performance of diagnostic tests via vs. was satisfactory with relatively high accuracy values. Interestingly, different groups have shown similar results with similar spectral and analytical methods. For oral cancer, Jaychandran et al. [14] and Rekha et al. [15] obtained a diagnostic accuracy of 93.1% and 89.1%, respectively, using the same pre-analytical conditions and PCA-LDA.

In contrast and surprisingly enough, for Sjögren’s syndrome (SjS), the same group found different results with an equivalent number of patients, the same technique (SERS), and an identical prediction model (PCA-LDA followed by LOOCV). Indeed, in 2019 Stefancu et al. obtained a sensitivity of 96.5%, specificity of 90.5%, and accuracy of 94% [28], while in 2020 Moisoiu et al. obtained a sensitivity of 77%, specificity of 74%, and accuracy of 75% [29]. These different performances could be related to the sample preparation method (deproteinized saliva) [28] or not [29]. Furthermore, the same group of researchers, in 2014, Feng et al. [16] obtained an accuracy value of 90.2% and Qiu et al. in 2016 [17], and accuracy value of 83.9% for nasopharynx cancer, despite an identical procedure for statistical analysis (PCA-LDA with LOOCV) and using the same approach as SERS. Again, the differing results could probably be explained by a change in the saliva preparation, the first group using total purified protein saliva, the second, frozen total saliva without cells, although the difference in the sample size may also impact on the accuracy values.

In addition, the salivary composition can be influenced by the collection time (as cortisol secretion peak between 06:00 to 08:00 a.m.), collection methods (stimulated or not), current medical treatments, the presence of comorbidities or oral inflammation [11,12]. In this review, for example, the time of sampling is not always specified in the included studies (*n* = 10/18), as well as the volume of saliva that was collected, varying from 1 to 4 mL when specified. The pre-analytical parameters are important to consider and can constitute a methodological bias. To our knowledge, there are no publications or guide specifying the importance of the pre-analytical preparation of saliva used in VS. However, for blood sampling, it has been shown that storage at −80 °C and in plastic tubes has no effect on generated spectra, whereas variations in the drying and the storage of samples (fresh or frozen, avoid freeze-thaw cycles) can have an effect [49]. Therefore, standardization of saliva sample handling (collection, processing, and storage protocols) is crucial to ensure reproducibility and consistency in the results obtained as suggested by other reports [4,18,50].

The concept of confounding factors is also very important, and yet, in all selected studies of this review, it is very inadequately addressed. It is difficult to know whether biases in patient selection exist, for example, with respect to age, sex, comorbidities, tobacco and alcohol consumption, drug uptake before/after saliva collection, as these factors may strongly influence the saliva content, and hence, its resulting spectral profile. Derruau et al. recently showed using saliva-based IR spectroscopy, that the periodontal diseases, a multifactorial inflammatory, and infectious oral disease could influence the salivary infrared spectra in the spectral range of lipids and proteins absorption (2800–3000 cm^−1^) [11] and could become a confounding factor in the detection of other multifactorial inflammatory diseases. Indeed, for Hernandez et al., in three different reports published in 2017 and 2019 [25,26,27], periodontal disease and breast cancer were discriminated by SERS using the same marker bands of sialic acid. Thus, one pathology becomes a confounding factor of the other, and *vice* versa. In Hernandez’s first publication on breast cancer, the patient inclusion criteria were based on information, such as ‘Patients had no oral complaints’. In the second, patients should not have periodontitis or gum bleeding. However, in both of these, there is no mention of who and how were these criteria evaluated. No specialists in oral diseases are in the author list. The implication of clinicians in connection with the development of clinical tests and particularly with respect to the concerned pathology is obvious to establish the validity of these clinical tests (e.g., in the assessment of patient inclusion criteria). Twenty-two percent of the publications cited in this review (*n* = 4/18) do not present any clinicians related to the studied pathology among the authors.

Other criteria are to be taken into account as the type of vs. used as analytical methods.

In this review, selected studies on saliva using IR, Raman, and SERS, respectively, gave accuracy values in the ranges 88.2–88.8%, 89.1–95.6%, and 70.5–94%. ATR-FTIR spectroscopy is very applicable to the routine monitoring of biofluids [51], but the drying of the sample is necessary, inducing a longer pre-analytical preparation time, which may however limit its clinical application. As water is a weak scatterer, Raman microspectroscopy is unaffected by aqueous solutions, permitting in vivo and live-cell imaging [52] and particularly amenable to saliva samples. Thus, in this review, this could explain the higher number of Raman studies (*n* = 16) compared to IR (*n* = 2).

However, it is difficult to conclude that one technique can perform better than the other, although in the case of IR, the drying process of the sample can introduce chemical and physical inhomogeneities in the sample, due to the so-called “coffee ring” effect, cracking and gelation patterns, that could impact on the reproducibility and sensitivity [53,54,55]. The comparison between these complementary vs. techniques was difficult in this review as the included studies were hardly comparable: (i) Only one study per disease was found for diabetes [30], gastric cancer [33], acute myocardial infarction [31], or periodontitis [27], (ii) the number of included patients is low, and (iii) only one group, Maitra et al., has used both techniques on the same set of saliva samples in the case of esophageal cancer. If the criterium *n* ≥ 20 is taken into account, only the control, Barrett’s esophagus, and OAC groups are potentially useful, with accuracy values of 88.8% and 95.6% for IR and Raman, respectively, using SPA QDA data processing.

In addition, in 2010, Scott et al. obtained quite similar IR results as Maitra et al. [21] for diabetes with an accuracy of 88.2% IR. On the other hand, if one considers the study by Caixeta et al., in 2020 on rats (*n* = 21), an accuracy of 95.2% and sensitivity of 100% were achieved [56]. The publication of robust larger studies is paramount for a proper comparison of the different techniques. More recently, Parachalil et al., undertook a similar comparison of Raman and ATR-FTIR of plasma using identical sample preparation and analysis protocols, to quantitatively monitor diagnostically relevant changes of glucose [57]. They demonstrated that liquid Raman spectroscopy can perform at least, as well as ATR-FTIR, which requires a drying step.

Based on the adsorption of molecules of the sample on metallic nanoparticles (NPs), the SERS approach has been developed to significantly increase the signal intensity (of the order of 10^5^ to 10^6^), as well as to decrease any sample autofluorescence [58]. By exploiting NPs, such as silver (Ag) or gold (Au), enhanced spectra are generated to allow a better characterization, detection, and identification of biomolecular analytes in a shorter timeframe. This explains the higher number of SERS studies (*n* = 12/18) in the selected articles. Yet, the intensity and shape of a SERS spectrum strictly depend on the combination of many experimental conditions, and the SERS effect could also be influenced by many factors, like laser power, temperature, solvent, SERS substrate, the ratio between the total number of nanoparticles and the volume of biofluid, but also on the stability of the NPs, the state of the analyte, and exposure time. All these factors could explain the disparities in the accuracy results obtained between 70.5% and 94%, whatever the studied pathologies. These are not significantly very different from values obtained with conventional Raman spectroscopy in the selected articles (from 89.1% to 95.6%).

Furthermore, it is also important to note that the choice of the data pre-processing and processing methods is also an important factor to be considered as they form part of the pre-analytical parameters. In 2014 and 2017, Feng et al. and Lin et al., from the same research group, reported accuracy values of 90.2% for 62 cancer and 30 healthy patients and 70.5% for 170 cancer and 71 healthy individuals using SERS and PCA-LDA-LOOCV for data analysis and the same saliva preparation. These results again show the importance of sample size, but also that data processing with a small number of patients may lead to an overestimation of the performances of the classification model. These models often require splitting into a training set, a validation set, and an independent prediction set, thus requiring a significantly high number of patients initially.

Another important aspect is the ROC curve analysis that is widely considered as the most objective and statistically valid method for evaluating biomarker performance, particularly in the context of clinical test development [16]. However, out of these 18 publications, only eight studies published by three teams used ROC curves with accuracy values ranging from 70.5% to 92%. In 2015, Feng et al. reported an accuracy value of 78.4% for breast cancer using SERS and PLS-DA-LOOCV associated with ROC curve, while Hernandès et al. in 2017 obtained an accuracy of 92%. It can be noted that Feng et al. based their classification model on 17 discriminant wavenumbers while Hernandès et al. exploited only three specific wavenumbers corresponding to sialic acid that is considered as a saliva biomarker for oral cancer [59]).

During the past years, single-molecule studies using SERS have been developed with the aim of quantifying the molecule implicated in the studied pathology. Indeed, in 2017, Hernandez et al. demonstrated the ability of SERS to measure concentrations of sialic acid in human saliva and to discriminate healthy from breast cancer patients with an accuracy of 92% [25]. Yet, in 2019, Hernandez et al., used the same technique and same discriminant frequencies, to delineate control patients from those with periodontitis, with an accuracy of 88.8% [26]. Although the good accuracy values indicate a high-performance classifier, these results raise several remarks. For instance, the same three marker bands related to sialic acid identified two different pathologies at two different anatomical sites, in breast cancer and in inflammatory disease of infectious origin (periodontitis). It appears that sialic acid does not represent a discriminating molecule in the evaluation of these two pathologies by SERS.

Furthermore, Stefenelli et al. reported that sialic acid levels are increased in the serum of patients with uterus, lung, colon/rectum, stomach, or prostate cancer. This may be indicative of the presence not only of breast cancer, but also of other types of cancers and/or cancer unrelated severe inflammatory conditions [60]. Sialic acid used alone can be a confounding factor, which confirms the importance of the selection of patients included by a history and collection of precise clinical data. A clinical test based on a number of discriminating bands should become more specific for the sought pathology. In the majority of the selected studies (*n* = 15/18), tentative assignments suggest the presence of several bands corresponding to proteins and amino acids (*n* = 7/18), lipids (*n* = 5/18), and DNA and/or RNA bases (*n* = 4/18). Lipid rich features in normal conditions and prominent protein features in tumors and other pathological conditions have been described. Among all these studies, some molecules more often appear to discriminate between healthy and diseased patients. In particular, the amide I band was assigned as a discriminant in 14 out of 18 studies, phenyl vibrations in 10 studies, Amide III, phenylalanine, tyrosine in eight studies, and tryptophan, proline in seven studies. Further, several research works have been reported on the profile of amino acids of human saliva and their use in disease diagnosis [61,62]. The varying levels of these amino acids in saliva, related to the degradation of proteins present in saliva, represent interesting markers for pathology detection [15].

In fact, the access to multiple biomarkers rather than single or few biomarkers would be better for patient care, in particular for screening patients at high risk or at an early stage of the disease [4]. Indeed, some selected studies in this review do not only include healthy or diseased patients, but also patients with precancerous lesions (oral and esophageal; *n* = 4), benign lesions (breast; *n* = 1), differentiate between early and late stages of cancer (gastric; *n* = 1) or superficial or deep damage (periodontal; *n* = 1). Early detection and prevention are the key strategies to manage cancer and intervenes at an early stage, therefore significantly reducing morbidity and mortality from the malignant disease [32]. There is a continuing effort in the search of new technology that can detect early biochemical signs of malignancy, and therefore, respond to these objectives. Using Raman spectroscopy, Jaychandra’s study revealed an efficient classification with an accuracy of 93.1% for saliva samples between normal, precancerous, and oral squamous cell carcinoma patients [14]. The biological components, pyrimidine, glycoproteins especially mucin, oxygenated hemocyanin, and carotenoids showed differences in the three groups of saliva, normal (*n* = 21), premalignant (oral leukoplakia, oral submucous fibrosis, (*n* = 87)), and malignant (oral squamous cell carcinoma, (*n* = 50)). Particularly, the peaks at 752 cm^−1^ of oxygenated hemocyanin, at 1158 and 1525 cm^−1^ of carotenoids in saliva show variations between the three groups. In 2017, Rekha et al., also succeeded using Raman spectroscopy, to separate control from precancerous and from malignant lesions of the same cancer type with a performance of 82.4% and 89.1%, respectively, in cross-validated groups [15]. The intensity of the amide band I was higher for malignancies than for pre-malignancies or normal patients, while the opposite was observed for the lipids intensity bands (1128, 1310, and 1742 cm^−1^). However, the accuracy value calculated from a normal group versus premalignant and malignant groups was only 55.4% The premalignant patient group with malignant patients in 25% of cases and 21.4% with control ones while malignant patients group with premalignant ones in 37.5% of cases. The study by Maitra et al. is even more revealing of the problem of choice and of the number of patients per group, and thus, failed to discriminate between premalignant and normal stages [21]. This finding underscores the need for larger-scale studies or for using alternative spectral data processing methods.

The validation of a new clinical diagnostic method, a long-term follow-up, and correlation with gold standard endpoints is primordial. To be accepted in routine practice, sensitivity, specificity, and accuracy values for disease diagnosis need to be exceptional, as is the ability to determine emerging or progressive diseases. The use of saliva-based vs. could become complementary to radiological, biological, histological investigations.

For breast core biopsy, the histopathological examination has reported sensitivity values between 90.1 and 93% and is more operator-dependent, while publications referred to in this review report accuracy values of 78.4 to 92% depending on the analytical methods used. For oral cancers, the diagnostic gold standard is clinical examination followed by a biopsy for histopathological confirmation, with accuracy values ranging from 75 to 90% [63,64].

This range can be explained, among other things, by the method of taking the biopsy, but is also very operator dependent. In this review, for this same pathology, Jaychandran et al. and Rekha et al. obtained accuracy values of 93.1 and 89.1%, respectively, using Raman spectroscopy. [14,15]. In addition, for lung cancers, the initial gold standard is the clinical examination associated with the chest X-ray with an accuracy of 81%, according to Quekel et al. 1999 [63]. In this review, for the diagnosis of lung cancer by salivary VS, the accuracy value obtained was 80% [19].

In summary, the accuracy values obtained by saliva-based vs. in comparison with those of gold-standard diagnostics, are quite comparable for cancer, results also found with other non-cancer pathologies, such as SjS [28,29,65]. Overall, the reported diagnostic measures for saliva-based vs. are promising with existing diagnostic modalities. Although diagnostic accuracy levels are high for VS, it is difficult to replace gold standards. However, the advantages of saliva-based vs. are numerous over, for example, invasive, painful, and complication-risk biopsies, radiography, and CT-induced irradiations. Saliva-based vs. is non-invasive, painless, with minimal or no sample preparation, no labeling, extemporaneous, quick, and easy to perform. In addition, saliva is a complex biofluid reflecting the physiological and pathological state of the body, due to the presence of numerous biocomponents. Saliva-based vs. could become a diagnostic tool complementary to the gold standard by detecting and potentially quantifying metabolites induced by the pathology and its evolution. Therefore, this technology could be used through a large range of clinical situations: screening of patients at risk of the disease or with the disease at an early stage, differential diagnosis of the disease with other conditions, the prognosis of the disease independently of the treatment, prediction of the response to treatment, and monitoring of disease progress [4,66].

Saliva is also comparable to human serum metabolomes in terms of chemical complexity and abundance of metabolites [9,12]. Also, vs. of saliva could be an alternative to vs. using blood or its derived products, without being invasive and stressful for the patient and not requiring a more complex storage mode. In this review, only three reports compared saliva with blood as sampling media. In the case of oral cancers, Jaychandan et al. obtained accuracy values of 91.3% and 78% for saliva and blood, respectively, using Raman spectroscopy [14]. In the case of esophageal cancers, Maitra et al. obtained accuracy values of 95.6%, 82.6%, and 91.3% for saliva, plasma, and serum, respectively [21].

The use of saliva-based vs. is promising in comparison with blood-based vs. and may appear more adequate also in the case of oral cancers, which are in direct contact with this biofluid.

## 4. Material and Methods

This study was conducted following the Preferred Reporting Items for Systematic Reviews and Meta-analyses (PRISMA) guidelines [67].

### 4.1. Research Question/Focused Question

This systematic review aimed at appreciating if Vibrational Spectroscopy could be applied to saliva samples as a disease diagnostic tool.

### 4.2. Search Strategy

Two electronic databases were searched: MEDLINE (PubMed, Public access to Medline) and Scopus. The last research was conducted on 30th May 2020, and any publication to this date was evaluated for inclusion. Keywords sentences for both databases are described in Table 3. Studies obtained for screening were downloaded into the Zotero research tool, duplicates were then identified and excluded from the total list of articles.

### 4.3. Inclusion and Exclusion Criteria/Eligibility Criteria/Study Selection Criteria

The PICOS framework (Population, Intervention, Comparison, Outcome, Study design process with added qualitative search terms) was used to set inclusion and exclusion criteria. Details of eligibility and exclusion criteria of studies are shown in Table 4.

Only publications with both a clinically and/or a histopathologically confirmed disease and a control group, each containing more than 20 participants, were included [40,68]. Studies involving non-saliva, non-human, animals, tissue samples, or pooled cells were excluded. Studies had to report characteristic parameters of the diagnostic tool, such as sensitivity, specificity, accuracy, or AUC of ROC curve.

### 4.4. Screening for Eligibility/Inclusion

Articles identified with keywords from MEDLINE-PubMed and Scopus search were screened by two members of the review team based on title and abstract according to inclusion and exclusion criteria. Potential divergences were solved by discussion. A full-text review of the selected articles was then performed by the same two members. The references of these articles were also checked to include any interesting papers that were not picked up during direct MEDLINE-PubMed and Scopus search. If relevant, these new publications were also downloaded and added to the list of full-text articles for assessment. Eligible articles were finally included in the systematic review.

### 4.5. Outcomes and Data Extraction

The outcomes related to the ability of vs. to diagnose diseases by analysis of human saliva included: sensitivity, specificity, accuracy, and AUC values.

Data were extracted from each article and stored in Excel^®^ (Microsoft, Redmond, Washington, USA) format. When multiple spectral techniques or data analysis techniques were evaluated within a study, data were described based on the most effective technique used. When data were presented for both a training set and test/cross-validation set, data from the test set were presented as these reflect most closely the performance of the test in clinical practice.

## 5. Conclusions

Saliva-based vs. appears promising, as it is based on the abilities to objectively fingerprint the biochemical profile underlying the early onset of disease. However, the studies included in this review lack robustness and are hardly comparable, which may further explain the divergence of the results and that a meta-analysis on this subject is currently not feasible. Furthermore, several parameters, such as the use of different substrates, laser frequency, detectors, temperatures, sample solvents, and others (pre-analytical conditions, data processing, etc.) impact on the performances of vs. techniques and could be a hindrance for routine clinical translation in the near future. Moreover, new methodological and technical strategies need to be developed to improve the reproducibility and the “standardization” of VS.

The recent years have evidenced the emergence of high-throughput screening (HTS) VS. techniques capable of providing rapid data collection, quality control, and classification processes. These approaches could indeed be promising for future saliva-based diagnostics approaches. Based on ATR-FTIR platform technology, the ClinSpec Dx™ spectroscopic liquid biopsy (blood), able to identify brain cancer disease at an early stage, is one of the first portable vs. applied in clinical practice. Furthermore, fiberoptic probes and miniaturization of instruments are also interesting for real-time and routine diagnosis. Interestingly, DIAFIR, a medtech company, has developed NASHMIR^®^, a non-invasive test based on the metabolic signature of NASH, from a simple drop of serum. This technology combines mid-IR technology with an ATR-based optical fiber biosensor. These portable tools could be particularly adapted to saliva for clinical applications. Concerning the SERS technology, there has been an ongoing development of SERS substrates—especially those involving gold- or silver-NPs in order to increase the reproducibility and enhance significantly molecule detection. The approach will require further refinement and substrate cost reduction for clinical application.

Taken together, although promising, further work is required before saliva-based vs. diagnostics could be confirmed, especially on larger cohorts, and translated to routine clinical use. Efforts should be ongoing to standardize saliva-based VS, taking into account pre-analytical and analytical requisites, prior to its development as a diagnostic/screening test for human diseases.

## Figures and Tables

**Table 1:** Studies included in the systematic literature review using salivary vibrational spectroscopy as a diagnostic tool for various diseases.

Diseases	VS Technique	Authors	Year	Number of Patients Included	Algorithm	Spectral Range (in cm^−1^)	Sensibility	Specificity	Accuracy
Oral squamous cell carcinoma	Raman	Jaychandran S. et al. [14]	2016	50 Cancers/87 Premalignant lesions/21 Healthy	PCA-LDA	600 to 1800	-	-	93.1%
	Raman	Rekha P. et al. [15]	2016	32 Cancers/28 Premalignant lesions/23 Healthy	PCA-LDA-LOOCV	800 to 1800	93.8%	82.6%	89.1%
Nasopharynx cancer	SERS	Feng S. et al. [16]	2014	62 Cancers/30 Healthy	PCA-LDA-LOOCV + ROC curve	500 to 1750	98.4%	73.3%	90.2%
	SERS	Qiu S. et al. [17]	2016	32 Cancers/30 Healthy	PCA-LDA-LOOCV + ROC curve	400 to 1750	86.7%	81.3%	83.9%
	SERS	Lin X. et al. [18]	2017	170 Cancers/71 Healthy	PCA-LDA-LOOCV + ROC curve	600 to 1750	70.7%	70.3%	70.5%
Lung cancer	SERS	Li X. et al. [19]	2012	21 Cancers/20 Healthy	PCA-LDA	500 to 2000	78%	83%	80%
	SERS	Qian K. et al. [20]	2018	61 Cancers/66 Healthy	Random Forest * (SVM -LOOCV)	400 to 1800	96.7%	100%	-
Œsophagal cancer	ATR-FTIR	Maitra I. et al. [21]	2019	25 OAC/12 HGD/6 LGD/27 Barrett’s/19 Esophageal inflammatory/38 Healthy 24 OAC/10 HGD/5 LGD/26 Barrett’s/18 Esophageal inflammatory/35 Healthy	SPA-QDA * (PCA-QDA, GA-QDA)	900 to 1800	95.4% ^#^	62.5% ^#^	88.8% ^#^
	Raman	Maitra I. et al. [22]	2020		SPA-QDA * (PCA-QDA, GA-QDA)	800 to 1800	100% ^#^	80% ^#^	95.6% ^#^
Gastric cancer	SERS	Chen Y. et al. [23]	2018	84 Late cancer/20 Early cancer/116 Healthy	PCA	Amino acids (400–2000)	87.7% ^#^	80% ^#^	-
Breast cancer	SERS	Feng S. et al. [24]	2015	31 Cancers/33 Benign tumor/33 Healthy	PLS-DA-LOOCV + ROC curve	500 to 1780	72.7% ^#^	81.3% ^#^	78.4% ^#^
	SERS	Hernández-Arteaga A. et al. [25]	2017	100 Cancers/106 Healthy	ROC curve analysis	Sialic acid (400–1800)	94%	98%	92%
	SERS	Hernández-Arteaga A. et al. [26]	2019	35 Cancers/129 Healthy	ROC curve analysis	Sialic acid (400–1800)	80.6%	93.1%	-
Periodontitis	SERS	Hernandez-Cedillo A. et al. [27]	2019	33 Periodontitis/30 Gingivitis/30 Healthy	ROC curve analysis	Sialic acid (400–1800)	69.6%	100%	-
Sjögren’s syndrome (SjS)	SERS	Stefancu A. et al. [28]	2019	29 SjS/21 Healthy	PCA-LDA-LOOCV	500 to 1750	96.5%	90.5%	94%
	SERS	Moisoiu V. et al. [29]	2020	31 SjS/22 Healthy	PCA-LDA-LOOCV	600 to 1700	77%	74%	75%
Diabetes	FTIR	Scott D.A. et al. [30]	2010	39 Diabetes/22 Healthy	LDA-cross validation	900 to 1800	90.9%	-	88.2%
Myocardial infarction (AMI)	Raman	Cao G. et al. [31]	2015	46 AMI/43 Healthy	PCA-LDA-LOOCV + ROC curve	400 to 1800	80.4%	81.4%	-

SERS, surface-enhanced Raman scattering; ATR-FTIR, attenuated total reflectance-Fourier transform infrared; HGD, high-grade dysplasia; LGD, low-grade dysplasia; OAC, esophageal adenocarcinoma; SjS, Sjögren’s syndrome; AMI, acute myocardial infarction. * results from the most relevant algorithm according to the authors; ^#^ minimal value according to the different categories of patients (≥20 patients).

**Table 2:** Molecular biosignatures and tentative assignments identified from saliva using FTIR, Raman, and SERS to diagnose different diseases.

Diseases	Authors	Year	VS Technique	Nature of Substrate (for SERS only)	Peak Wavenumbers (in cm^−1^)	Major Assignments
Oral squamous cell carcinoma	Jaychandran S. et al. [14]	2016	Raman	-	767, 1236, 1330, 1662, 1688	Pyrimidine
					1652	Amide
					1444	Mucine
					752	Hemocyanine
	Rekha P. et al. [15]	2016	Raman	-	806, 1460, 1485	DNA (O-P-O symmetric stretch, Pentose sugar CH_2_ deformation vibration, Purine base vibration)
					829, 1142, 1169, 1660	Glutathione
					870, 896, 986	Proline (C-C stretch, na, na)
					918	Histidine
					935, 948, 964, 969	Valine (C-C stretch, na, na, na)
					1015, 1338, 1360, 1424, 1556	Tryptophan (benzene and pyrrole ring breathe out of
					1050, 1090	phase, Fermi resonance doublet, na, na)
					1066, 1128, 1302, 1735	Lactic acid (C–CH_3_ stretch, C–O stretch)
					1509	Lipid (na, C-C stretch, CH_2_ twisting and wagging, C=O stretch)
					1180	Phenylalanine
					1238, 1258, 1276	Tyrosine, cytosine, guanine, adenine
					1636	Amide III (C-N stretch)
					806, 1460, 1485	Amide I (C=O stretch)
Nasopharynx cancer	Feng S. et al. [16]	2014	SERS	Ag-Colloids	621, 1004, 1031	Phenylalanine (C-C twisting mode, ν_s_(C-C), δ(C-C))
					642, 1173	Tyrosine (ν(C-S))
					760	Tryptophan (ring breathing mode)
					933	Proline (ν(C-C))
					1123	Proteins (ν(C-N))
					1337	Collagen (CH_3_CH_2_ wagging)
					1445	Collagen, phospholipids (δ(C-H))
	Qiu S. et al. [17]	2016	SERS	Ag-Colloids	447, 1003	Phenylalanine (Ring torsion, ν_s_(C-C))
					496	Glycogen
					590	Ascorbic acid, Amide VI
					635	L-Tyrosine, Lactose (ν(C-S))
					725	Adenine, Coenzyme A (δ(C-H))
					812	L-Serine (ν(C-C-O))
					888	D-Galactosamine (δ(C-O-H))
					1052	Protein (C-O/C-N stretching)
					1134	D-Mannose (ν(C-N))
					1204	L-Tryptophane, Phenylalanine (Ring vibration)
					1270	Unsaturated fatty acids (ν(C-H))
					1336	Nucleic acid bases (ν(C-H))
					1448	Collagen, phospholipids (δ(CH_2_))
					1619	Tryptophan (ν(C=C))
					1662	Nucleic acid
	Lin X. et al. [18]	2017	SERS	Ag-Colloids	621, 1004, 1031	Phenylalanine (C-C twisting mode, ν_s_(C-C), δ(C-H))
					642, 854, 1175	Tyrosine (ν(C-S), Ring breathing mode, δ(C-H))
					760, 1208, 1552	Tryptophan (Ring breathing mode, ν(C-C_6_H_5_), ν(C=C))
					878	Hydroxyproline (ν(C-C))
					935	Proline (ν(C-C))
					959	α-helix Proline, Valine (ν(C-C))
					1049, 1123	Proteins (ν(C-O) ν(C-N), ν(C-N))
					1265	Amide III, collagen (ν(CN), δ(NH))
					1337	Collagen (CH_3_CH_2_ wagging)
					1445	Collagen, lipids
					1684	Amide I (ν(C=C))
Lung cancer	Li X. et al. [19]	2012	SERS	Ag-Colloids	523	Lysozymes, proteins, guanine, thymine
					622	Proteins, phenylalanine, adenine
					696	Methionine, cytosine
					735	Tryptophan, coenzyme A, adenine, cytosine, thymine, guanine
					789	Cytosine, uracil, thymine
					822	-
					884	Proline, valine, glycine, tryptophan, glutamic acid, hydroxyproline
					909	Tyrosine
					925	Proline, glucose
					1009	Tryptophan, lysine, phenylalanine
					1077	Lipids, nucleic acids, proteins, carbohydrates
					1280	Phospholipid, amide III, proteins, lipids
					1369	Tryptohan, porphyrins, lipids, guanine, thymine, proteins
					1393	-
					1722	Ester group
	Qian K. et al. [20]	2018	SERS	Gold nano-modified chip (OptoTrace Technologies)	423	Glucose, deuterated glucose
					643	(C-H torsion, COO^-^ wag; O-C=O in plane deformation; C-C-C in phase deformation)
					672	Cytosine, guanine (C–S stretch)
					732	Adenine (C–S (protein)/CH2 rocking)
					852	Tyrosine (Ring breathing mode), Proline Ring (C–C stretch)
					923	Proline Ring (C-C stretch), Lactic Acid, glucose
					999	Phenylalanine (symmetric ring breathing mode)
					1030	(Stretching vibration of the ring, deformation in plane C-H)
					1046	N-acetyl glucosamine
					1268	Amide III (C–N stretching mode of proteins, indicating mainly a-helix conformation)
					1449	Phenylalanine, Proteins (CH_2_ bending mode), Bending mode (C=C)
					1600	Phenylalanine, Tyrosine (C=C in-plane bending mode)
Œsophagal cancer	Maitra I. et al. [21]	2019	ATR-FTIR		902	Phosphodiester region
					991	Ribose (C-O), (C-C)
					1003	(Ring stretching vibrations mixed strongly with CH in-plane bending)
					1014, 1107	Polysaccharides, pectin (ν(CO), ν(CC), δ(OCH), ring)
					1068	Ribose (Stretching C-O)
					1099	Phosphate II (Stretching PO_2_^-^ symmetric)
					1431	Polysaccharides, cellulose (δ(CH_2_))
					1558	(Ring base)
					1589	Phenyl (Ring C-C stretch)
					1604	Adenine (DNA)
					1624, 1689	Nucleic acids (base carbonyl stretching, ring breathing mode)
					1643	Amide I (C=O stretching vibrations)
					1697, 1701	Guanine (C_2_=O, C_5_=O)
					1716	Thymine (C=O)
					1743	Lipids (C=O stretching mode)
					1778, 1786	Lipids (ν(C=C), ν(C=C)), fatty acids
	Maitra I. et al. [22]	2020	Raman		902	Phosphodiester region
					991, 1068	Ribose (C-O), (C-C)
					1003	(Ring stretching vibrations mixed strongly with CH in-plane bending)
					1014, 1107	Polysaccharides, pectin (ν(CO), ν(CC), δ(OCH), ring)
					1068	Ribose (Stretching C-O)
					1099	Phosphate II (Stretching PO_2_^-^ symmetric)
					1431	Polysaccharides, cellulose (δ(CH_2_))
					1558	(Ring base)
					1589	Phenyl (Ring C-C stretch)
					1604	Adenine (DNA)
					1624, 1689	Nucleic acids (base carbonyl stretching, ring breathing mode)
					1643	Amide I (C=O stretching vibrations)
					1697, 1701	Guanine (C_2_=O, C_5_=O)
					1716	Thymine (C=O)
					1743	Lipids (C=O stretching mode)
					1778, 1786	Lipids (ν(C=C), ν(C=C)), fatty acids
Gastric cancer	Chen Y. et al. [23]	2018	SERS	A/GO NSs	435	Glutamine, hydroxylysine, proline, tyrosine
					488	Taurine, glycine, ethanolamine, hydroxylysine, tyrosine
					530	Taurine, glutamine, histidine, alanine, glutamic acid
					642	Histidine, alanine, proline, tyrosine
					725	Taurine, glutamine, histidine, glutamic acid
					781	Glycine, glutamic acid, proline, tyrosine
					843	Taurine, ethanolamine, histidine, alanine, hydroxylysine, proline, tyrosine
					869	Glycine, glutamine, ethanolamine, glutamic acid
					917	Glutamine, alanine, glutamic acid, proline
					933	Histidine, glutamic acid, proline
					961	Histidine, glutamic acid, proline, tyrosine
					1037	Taurine, ethanolamine, alanine, proline, tyrosine
					1053	Taurine, glutamine, ethanolamine, hydroxylysine
					1109	Taurine, glutamine, ethanolamine, histidine, alanine
					1197	Histidine, hydroxylysine, proline, tyrosine
					1222	Hydroxylysine, proline, tyrosine
					1450	Taurine, glycine, glutamine, ethanolamine, alanine, glutamic acid, hydroxylysine, proline
					1500	Histidine
					1710	Glutamine
Breast cancer	Feng S. et al. [24]	2015	SERS	Ag-Colloids	621, 643, 1004, 1033	Phenylalanine (C-C twisting mode, C-C twisting mode, ν_s_(C-C), δ(C-H))
					760, 1208, 1552	Tryptophan (Ring breathing mode, ν(C-C_6_H_5_), ν(C=C))
					854, 1176	Tyrosine (Ring breathing mode, δ(C-H))
					876	Hydroxyproline (ν(C-C))
					935	Proline (ν(C-C))
					1049, 1084	Proteins (ν(C-O) ν(C-N), ν(C-N))
					1265	Amide III, collagen (ν(CN), δ(NH))
					1340	Collagen (CH_3_CH_2_ wagging)
					1447	Collagen, Lipids (δ(C-H))
					1684	Amide I (ν(C=C))
	Hernández-Arteaga A. et al. [25]	2017	SERS	Cit-Ag-NP	1002	Pyranose (Ring breathing mode)
					1237	Amide III (C-N stretching)
					1391	Carboxyl (stretching mode)
	Hernández-Arteaga A. et al. [26]	2019	SERS	Cit-Ag-NP	1002	Pyranose (Ring breathing mode)
					1237	Amide III (C-N stretching)
					1391	Carboxyl (stretching mode)
Periodontitis	Hernandez-Cedillo A. et al. [27]	2019	SERS	Cit-Ag-NP	1002	Pyranose (Ring breathing mode)
					1237	Amide III (C-N stretching)
					1391	Carboxyl (stretching mode)
Sjögren’s syndrome (SjS)	Stefancu A. et al. [28]	2019	SERS	Ag-NP	724, 1095, 1323, 1450, 1570	Hypoxanthine (na, R2trigd or bC-H (in-plane), C-O, C-N or C-C, C-N)
					956, 1134, 1245, 1323	Xanthine (bN-H, R2trigd, C-N, Ring vibrations, C-N, bC-H, C-N)
					884, 1130, 1370	Uric acid (na, na, na, C-N, C-H bending)
	Moisoiu V. et al. [29]	2020	SERS	Cl-Ag-NP	724, 1097, 1324, 1449, 1581	Hypoxanthine (na, Ring vibrations, C-O, C-N or C-C, C-N)
					957, 1132, 1245, 1324	Xanthine (na, Ring vibrations, C-N, Mixed ring vibrations/C-N)
					812, 886, 1132, 1369	Uric acid (na, na, C-N, C-N, C-H bending)
					1002, 1032, 1205, 1651	Proteins (Phe, Phe, Try/Phe, Amide I)
Diabetes	Scott DA. Et al. [30]	2010	FTIR		≈970	(C-C/C-O stretching vibrations in sugar moieties)
					≈1150	(C-C/C-O stretching vibrations in sugar moieties, C-O-C symmetric and asymmetric vibrations of sugar moieties and phospholipids)
					≈1410	(v_s_(COO^−1^), symmetric and asymmetric carboxyl radical stretching vibrations of carboxylate groups)
					≈1470	(bending vibration of CH_2_ group of amino acids in protein side chains)
					≈1695	(α-helix component in the amide I region, intermolecular antiparallel b-sheets)
					≈1745	(lipid ester band)
Myocardial infarction (IMA)	Cao G. et al. [31]	2015	Raman		442	(N-C-S stretch)
					509	Cystein (ν(S–S) gauche–gauche–gauche)
					621, 1002, 1031	Phenylalanine (C–C twisting mode of phenylalanine, δ(C–H))
					643, 828, 853	Tyrosine (C–C twisting, Ring breathing tyrosine, Ring breathing mode of tyrosine)
					755	Tryptophan (ν(C–C))
					876	Hydroxyproline
					925	(C–H bending)
					1047	(C–CH_3_ vibration)
					1210	Hydroxyproline, Tyrosine
					1330	Nucleic Acids
					1449	Proteins (C–H vibration)
					1555	Amide II
					1670	Amide I

Cit-Ag-NP, citrate-reduced silver nanoparticles; Ag-NP, silver nanoparticles; Cl-Ag-Np, chloride-capped silver nanoparticles; A/GO NSs, graphene oxide nanoscrolls wrapped with gold nanoparticles; Ag-Colloids, silver colloids; ν, stretching; ν_s_, symmetric stretch; b, bending; R, ring; trigd, trigonal deformation; δ, deformation; na, not assigned. Tentative assignments are taken from cited publications.

**Table 3:** Search strategies.

MeSH Terms Used for MEDLINE Search	Keywords Used for Scopus Search
(“saliva”[MeSH Terms] OR saliva[Title/Abstract]) AND (“diagnosis”[MeSH Terms] OR diagnosis[Title/Abstract] OR “biomarkers”[MeSH Terms] OR biomarkers[Title/Abstract] OR diagnostic[Title/Abstract]) AND (“spectroscopy, fourier transform infrared”[MeSH Terms] OR “infrared spectroscopy”[Title/Abstract] OR “spectrum analysis, Raman”[MeSH Terms] OR “Raman”[Title/Abstract])	(saliva) AND (diagnosis OR biomarkers OR diagnostic) AND (“infrared spectroscopy” OR Raman)

**Table 4:** Criteria for inclusion and exclusion of studies in the systematic review.

Item	Criteria of Inclusion	Criteria of Exclusion
population and conditions of interest	human population with clinical signs of disease with or without histopathologically confirmed disease diagnosis	non-human study
intervention/exposure/investigation	application of vs. to the analysis of human saliva with the specific aim of disease diagnosis	method other than vs. used as the main method of analysis
comparison	diseased population versus healthy population as the control group	no control group
outcomes of interest	performance of diagnostic tool (sensitivity, specificity, accuracy)	n.s.
study design	any study design fitting the above criteria	study with less than 20 participants in each group (diseased and control)
type of paper	original paper. manuscript is written in English	review article, opinion, commentary abstract from a conference, or not a peer-reviewed article.

n.s., not stated.

## References

[1] Hood L., Friend S.H. (2011). Predictive, personalized, preventive, participatory (P4) cancer medicine. Nat. Rev. Clin. Oncol..

[2] Delli K., Villa A., Farah C.S., Celentano A., Ojeda D., Peterson D.E., Jensen S.B., Glurich I., Vissink A. (2019). World Workshop on Oral Medicine VII: Biomarkers predicting lymphoma in the salivary glands of patients with Sjögren’s syndrome—A systematic review. Oral Dis..

[3] Hodson R. (2016). Precision medicine. Nature.

[4] Baker M.J., Hussain S.R., Lovergne L., Untereiner V., Hughes C., Lukaszewski R.A., Thiéfin G., Sockalingum G.D. (2016). Developing and understanding biofluid vibrational spectroscopy: A critical review. Chem. Soc. Rev..

[5] Untereiner V., Dhruvananda Sockalingum G., Garnotel R., Gobinet C., Ramaholimihaso F., Ehrhard F., Diebold M.-D., Thiéfin G. (2014). Bile analysis using high-throughput FTIR spectroscopy for the diagnosis of malignant biliary strictures: A pilot study in 57 patients: Spectral diagnosis of malignant biliary strictures. J. Biophotonics.

[6] Gaydou V., Polette M., Gobinet C., Kileztky C., Angiboust J.-F., Manfait M., Birembaut P., Piot O. (2016). Vibrational Analysis of Lung Tumor Cell Lines: Implementation of an Invasiveness Scale Based on the Cell Infrared Signatures. Anal. Chem..

[7] Nallala J., Piot O., Diebold M.-D., Gobinet C., Bouché O., Manfait M., Sockalingum G.D. (2013). Infrared imaging as a cancer diagnostic tool: Introducing a new concept of spectral barcodes for identifying molecular changes in colon tumors. Cytometry A.

[8] Leal L.B., Nogueira M.S., Canevari R.A., Carvalho L.F.C.S. (2018). Vibration spectroscopy and body biofluids: Literature review for clinical applications. Photodiagn. Photodyn. Ther..

[9] Kaczor-Urbanowicz K.E., Martin Carreras-Presas C., Aro K., Tu M., Garcia-Godoy F., Wong D.T. (2017). Saliva diagnostics – Current views and directions. Exp. Biol. Med..

[10] Schafer C.A., Schafer J.J., Yakob M., Lima P., Camargo P., Wong D.T.W. (2014). Saliva diagnostics: Utilizing oral fluids to determine health status. Monogr. Oral Sci..

[11] Derruau S., Gobinet C., Mateu A., Untereiner V., Lorimier S., Piot O. (2019). Shedding light on confounding factors likely to affect salivary infrared biosignatures. Anal. Bioanal. Chem..

[12] Streckfus C.F. (2015). Advances in Salivary Diagnostics.

[13] Wang A., Wang C.P., Tu M., Wong D.T.W. (2016). Oral Biofluid Biomarker Research: Current Status and Emerging Frontiers. Diagnostics.

[14] Jaychandran S., Meenapriya P., Ganesan S. (2016). Raman Spectroscopic Analysis of Blood, Urine, Saliva and Tissue of Oral Potentially Malignant Disorders and Malignancy-A Diagnostic Study. Int. J. Oral Craniofac. Sci..

[15] Rekha P., Aruna P., Brindha E., Koteeswaran D., Baludavid M., Ganesan S. (2016). Near-infrared Raman spectroscopic characterization of salivary metabolites in the discrimination of normal from oral premalignant and malignant conditions: Near-infrared Raman spectroscopic characterization of salivary metabolites. J. Raman Spectrosc..

[16] Feng S., Lin D., Lin J., Huang Z., Chen G., Li Y., Huang S., Zhao J., Chen R., Zeng H. (2014). Saliva analysis combining membrane protein purification with surface-enhanced Raman spectroscopy for nasopharyngeal cancer detection. Appl. Phys. Lett..

[17] Qiu S., Xu Y., Huang L., Zheng W., Huang C., Huang S., Lin J., Lin D., Feng S., Chen R. (2016). Non-invasive detection of nasopharyngeal carcinoma using saliva surface-enhanced Raman spectroscopy. Oncol. Lett..

[18] Lin X., Lin D., Ge X., Qiu S., Feng S., Chen R. (2017). Noninvasive detection of nasopharyngeal carcinoma based on saliva proteins using surface-enhanced Raman spectroscopy. J. Biomed. Opt..

[19] Li X., Yang T., Lin J. (2012). Spectral analysis of human saliva for detection of lung cancer using surface-enhanced Raman spectroscopy. J. Biomed. Opt..

[20] Qian K., Wang Y., Hua L., Chen A., Zhang Y. (2018). New method of lung cancer detection by saliva test using surface-enhanced Raman spectroscopy. Thorac. Cancer.

[21] Maitra I., Morais C.L.M., Lima K.M.G., Ashton K.M., Date R.S., Martin F.L. (2019). Attenuated total reflection Fourier-transform infrared spectral discrimination in human bodily fluids of oesophageal transformation to adenocarcinoma. Analyst.

[22] Maitra I., Morais C.L.M., Lima K.M.G., Ashton K.M., Date R.S., Martin F.L. (2020). Raman spectral discrimination in human liquid biopsies of oesophageal transformation to adenocarcinoma. J. Biophotonics.

[23] Chen Y., Cheng S., Zhang A., Song J., Chang J., Wang K., Gao G., Zhang Y., Li S., Liu H. (2018). Salivary analysis based on surface enhanced Raman scattering sensors distinguishes early and advanced gastric cancer patients from healthy persons. J. Biomed. Opt..

[24] Feng S., Huang S., Lin D., Chen G., Xu Y., Li Y., Huang Z., Pan J., Chen R., Zeng H. (2015). Surface-enhanced Raman spectroscopy of saliva proteins for the noninvasive differentiation of benign and malignant breast tumors. Int. J. Nanomed..

[25] Hernández-Arteaga A., de Jesús Zermeño Nava J., Kolosovas-Machuca E.S., Velázquez-Salazar J.J., Vinogradova E., José-Yacamán M., Navarro-Contreras H.R. (2017). Diagnosis of breast cancer by analysis of sialic acid concentrations in human saliva by surface-enhanced Raman spectroscopy of silver nanoparticles. Nano Res..

[26] Hernández-Arteaga A.C., de Jesús Zermeño-Nava J., Martínez-Martínez M.U., Hernández-Cedillo A., Ojeda-Galván H.J., José-Yacamán M., Navarro-Contreras H.R. (2019). Determination of salivary sialic acid through nanotechnology: A useful biomarker for the screening of breast cancer. Arch. Med. Res..

[27] Hernández-Cedillo A., García-Valdivieso M.G., Hernández-Arteaga A.C., Patiño-Marín N., Vértiz-Hernández Á.A., José-Yacamán M., Navarro-Contreras H.R. (2019). Determination of sialic acid levels by using surface-enhanced Raman spectroscopy in periodontitis and gingivitis. Oral Dis..

[28] Stefancu A., Badarinza M., Moisoiu V., Iancu S.D., Serban O., Leopold N., Fodor D. (2019). SERS-based liquid biopsy of saliva and serum from patients with Sjögren’s syndrome. Anal. Bioanal. Chem..

[29] Moisoiu V., Badarinza M., Stefancu A., Iancu S.D., Serban O., Leopold N., Fodor D. (2020). Combining surface-enhanced Raman scattering (SERS) of saliva and two-dimensional shear wave elastography (2D-SWE) of the parotid glands in the diagnosis of Sjögren’s syndrome. Spectrochim. Acta A Mol. Biomol. Spectrosc..

[30] Scott D.A., Renaud D.E., Krishnasamy S., Meriç P., Buduneli N., Çetinkalp Ş., Liu K.-Z. (2010). Diabetes-related molecular signatures in infrared spectra of human saliva. Diabetol. Metab. Syndr..

[31] Cao G., Chen M., Chen Y., Huang Z., Lin J., Lin J., Xu Z., Wu S., Huang W., Weng G. (2015). A potential method for non-invasive acute myocardial infarction detection based on saliva Raman spectroscopy and multivariate analysis. Laser Phys. Lett..

[32] Bray F., Ferlay J., Soerjomataram I., Siegel R.L., Torre L.A., Jemal A. (2018). Global cancer statistics 2018: GLOBOCAN estimates of incidence and mortality worldwide for 36 cancers in 185 countries. CA Cancer J. Clin..

[33] Chen Y., Zhang J., Guo L., Liu L., Wen J., Xu L., Yan M., Li Z., Zhang X., Nan P. (2016). A characteristic biosignature for discrimination of gastric cancer from healthy population by high throughput GC-MS analysis. Oncotarget.

[34] Abate-Shen C., Shen M.M. (2009). The prostate-cancer metabolome. Nature.

[35] Ozturk L.K., Emekli-Alturfan E., Kasikci E., Demir G., Yarat A. (2011). Salivary Total Sialic Acid Levels Increase in Breast Cancer Patients: A Preliminary Study. Med. Chem..

[36] Caton J.G., Armitage G., Berglundh T., Chapple I.L.C., Jepsen S., Kornman K.S., Mealey B.L., Papapanou P.N., Sanz M., Tonetti M.S. (2018). A new classification scheme for periodontal and peri-implant diseases and conditions – Introduction and key changes from the 1999 classification. J. Clin. Periodontol..

[37] Xiang X., Duarte P.M., Lima J.A., Santos V.R., Gonçalves T.D., Miranda T.S., Liu K.-Z. (2013). Diabetes-Associated Periodontitis Molecular Features in Infrared Spectra of Gingival Crevicular Fluid. J. Periodontol.

[38] Baeza M., Morales A., Cisterna C., Cavalla F., Jara G., Isamitt Y., Pino P., Gamonal J. (2020). Effect of periodontal treatment in patients with periodontitis and diabetes: Systematic review and meta-analysis. J. Appl. Oral Sci..

[39] Reed G.W., Rossi J.E., Cannon C.P. (2017). Acute myocardial infarction. Lancet.

[40] Bell M.L., Whitehead A.L., Julious S.A. (2018). Guidance for using pilot studies to inform the design of intervention trials with continuous outcomes. Clin. Epidemiol..

[41] Rodrigues L.M., Magrini Alva T.D., da Silva Martinho H., Almeida J.D. (2019). Analysis of saliva composition in patients with burning mouth syndrome (BMS) by FTIR spectroscopy. Vib. Spectrosc..

[42] Zamora-Mendoza B.N., Espinosa-Tanguma R., Ramírez-Elías M.G., Cabrera-Alonso R., Montero-Moran G., Portales-Pérez D., Rosales-Romo J.A., Gonzalez J.F., Gonzalez C. (2019). Surface-enhanced raman spectroscopy: A non invasive alternative procedure for early detection in childhood asthma biomarkers in saliva. Photodiagn. Photodyn. Ther..

[43] Ralbovsky N.M., Halámková L., Wall K., Anderson-Hanley C., Lednev I.K. (2019). Screening for Alzheimer’s Disease Using Saliva: A New Approach Based on Machine Learning and Raman Hyperspectroscopy. J. Alzheimers Dis..

[44] Rodrigues R.P., Aguiar E.M., Cardoso-Sousa L., Caixeta D.C., Guedes C.C., Siqueira W.L., Maia Y.C.P., Cardoso S.V., Sabino-Silva R. (2019). Differential Molecular Signature of Human Saliva Using ATR-FTIR Spectroscopy for Chronic Kidney Disease Diagnosis. Braz. Dent. J..

[45] Zermeño-Nava J.d.J., Martínez-Martínez M.U., Rámirez-de-Ávila A.L., Hernández-Arteaga A.C., García-Valdivieso M.G., Hernández-Cedillo A., José-Yacamán M., Navarro-Contreras H.R. (2018). Determination of sialic acid in saliva by means of surface-enhanced Raman spectroscopy as a marker in adnexal mass patients: Ovarian cancer vs. benign cases. J. Ovarian Res..

[46] Eom G., Hwang A., Kim H., Yang S., Lee D.K., Song S., Ha K., Jeong J., Jung J., Lim E.-K. (2019). Diagnosis of Tamiflu-Resistant Influenza Virus in Human Nasal Fluid and Saliva Using Surface-Enhanced Raman Scattering. ACS Sens..

[47] Žukovskaja O., Jahn I.J., Weber K., Cialla-May D., Popp J. (2017). Detection of Pseudomonas aeruginosa metabolite pyocyanin in water and saliva by employing the SERS technique. Sensors.

[48] Malkovskiy A.V., Yacob A.A., Dunn C.E., Zirbes J.M., Ryan S.P., Bollyky P.L., Rajadas J., Milla C.E. (2019). Salivary Thiocyanate as a Biomarker of Cystic Fibrosis Transmembrane Regulator Function. Anal. Chem..

[49] Lovergne L., Lovergne J., Bouzy P., Untereiner V., Offroy M., Garnotel R., Thiéfin G., Baker M.J., Sockalingum G.D. (2019). Investigating pre-analytical requirements for serum and plasma based infrared spectro-diagnostic. J. Biophotonics.

[50] Calado G., Behl I., Daniel A., Byrne H.J., Lyng F.M. (2019). Raman spectroscopic analysis of saliva for the diagnosis of oral cancer: A systematic review. Translational Biophotonics.

[51] Dorling K.M., Baker M.J. (2013). Rapid FTIR chemical imaging: Highlighting FPA detectors. Trends Biotechnol..

[52] Patel I.I., Trevisan J., Evans G., Llabjani V., Martin-Hirsch P.L., Stringfellow H.F., Martin F.L. (2011). High contrast images of uterine tissue derived using Raman microspectroscopy with the empty modelling approach of multivariate curve resolution-alternating least squares. Analyst.

[53] Baker M.J., Byrne H.J., Chalmers J., Gardner P., Goodacre R., Henderson A., Kazarian S.G., Martin F.L., Moger J., Stone N. (2018). Clinical applications of infrared and Raman spectroscopy: State of play and future challenges. Analyst.

[54] Filik J., Stone N. (2008). Analysis of human tear fluid by Raman spectroscopy. Anal. Chim. Acta.

[55] Filik J., Stone N. (2007). Drop coating deposition Raman spectroscopy of protein mixtures. Analyst.

[56] Caixeta D.C., Aguiar E.M.G., Cardoso-Sousa L., Coelho L.M.D., Oliveira S.W., Espindola F.S., Raniero L., Crosara K.T.B., Baker M.J., Siqueira W.L. (2020). Salivary molecular spectroscopy: A sustainable, rapid and non-invasive monitoring tool for diabetes mellitus during insulin treatment. PLoS ONE.

[57] Parachalil D.R., Brankin B., McIntyre J., Byrne H.J. (2018). Raman spectroscopic analysis of high molecular weight proteins in solution–considerations for sample analysis and data pre-processing. Analyst.

[58] Kong K., Kendall C., Stone N., Notingher I. (2015). Raman spectroscopy for medical diagnostics—From in-vitro biofluid assays to in-vivo cancer detection. Adv. Drug Deliv. Rev..

[59] Achalli S., Madi M., Babu S.G., Shetty S.R., Kumari S., Bhat S. (2017). Sialic acid as a biomarker of oral potentially malignant disorders and oral cancer. Indian J. Dent. Res..

[60] Stefenelli N., Klotz H., Engel A., Bauer P. (1985). Serum sialic acid in malignant tumors, bacterial infections, and chronic liver diseases. J. Cancer Res. Clin. Oncol..

[61] Reddy I., Sherlin H.J., Ramani P., Premkumar P., Natesan A., Chandrasekar T. (2012). Amino acid profile of saliva from patients with oral squamous cell carcinoma using high performance liquid chromatography. J. Oral Sci.

[62] Sugimoto M., Wong D.T., Hirayama A., Soga T., Tomita M. (2010). Capillary electrophoresis mass spectrometry-based saliva metabolomics identified oral, breast and pancreatic cancer-specific profiles. Metabolomics.

[63] Chen S., Forman M., Sadow P.M., August M. (2016). The Diagnostic Accuracy of Incisional Biopsy in the Oral Cavity. J. Oral Maxillofac. Surg..

[64] Pentenero M., Carrozzo M., Pagano M., Galliano D., Broccoletti R., Scully C., Gandolfo S. (2003). Oral mucosal dysplastic lesions and early squamous cell carcinomas: Underdiagnosis from incisional biopsy. Oral Dis..

[65] Giovelli R.A., Santos M.C.S., Serrano É.V., Valim V. (2015). Clinical characteristics and biopsy accuracy in suspected cases of Sjögren’s syndrome referred to labial salivary gland biopsy. BMC Musculoskelet. Disord..

[66] Unertl K.M., Fair A.M., Favours J.S., Dolor R.J., Smoot D., Wilkins C.H. (2018). Clinicians’ perspectives on and interest in participating in a clinical data research network across the Southeastern United States. BMC Health Serv. Res..

[67] Moher D., Liberati A., Tetzlaff J., Altman D.G. (2009). PRISMA Group Preferred reporting items for systematic reviews and meta-analyses: The PRISMA statement. BMJ.

[68] Hertzog M.A. (2008). Considerations in determining sample size for pilot studies. Res. Nurs. Health.

